# Targeting the apelin system for the treatment of cardiovascular diseases

**DOI:** 10.1093/cvr/cvad171

**Published:** 2023-11-13

**Authors:** Fiona A Chapman, Janet J Maguire, David E Newby, Anthony P Davenport, Neeraj Dhaun

**Affiliations:** BHF/University of Edinburgh Centre for Cardiovascular Science, Queen’s Medical Research Institute, Edinburgh, UK; Department of Renal Medicine, Royal Infirmary of Edinburgh, Edinburgh, UK; Division of Experimental Medicine and Immunotherapeutics, Addenbrooke’s Centre for Clinical Investigation, University of Cambridge, Cambridge, UK; BHF/University of Edinburgh Centre for Cardiovascular Science, Queen’s Medical Research Institute, Edinburgh, UK; Department of Renal Medicine, Royal Infirmary of Edinburgh, Edinburgh, UK; BHF/University of Edinburgh Centre for Cardiovascular Science, Queen’s Medical Research Institute, Edinburgh, UK; Department of Renal Medicine, Royal Infirmary of Edinburgh, Edinburgh, UK

**Keywords:** Apelin, Inotrope, Vasodilator, Cardiovascular disease

## Abstract

Cardiovascular disease is the leading cause of death worldwide. Its prevalence is rising due to ageing populations and the increasing incidence of diseases such as chronic kidney disease, obesity, and diabetes that are associated with elevated cardiovascular risk. Despite currently available treatments, there remains a huge burden of cardiovascular disease-associated morbidity for patients and healthcare systems, and newer treatments are needed. The apelin system, comprising the apelin receptor and its two endogenous ligands apelin and elabela, is a broad regulator of physiology that opposes the actions of the renin-angiotensin and vasopressin systems. Activation of the apelin receptor promotes endothelium-dependent vasodilatation and inotropy, lowers blood pressure, and promotes angiogenesis. The apelin system appears to protect against arrhythmias, inhibits thrombosis, and has broad anti-inflammatory and anti-fibrotic actions. It also promotes aqueous diuresis through direct and indirect (central) effects in the kidney. Thus, the apelin system offers therapeutic promise for a range of cardiovascular, kidney, and metabolic diseases. This review will discuss current cardiovascular disease targets of the apelin system and future clinical utility of apelin receptor agonism.

## 1. Introduction

The global prevalence of cardiovascular disease has nearly doubled in the last 30 years, and mortality is rising. In 2019, 18.6 million deaths were attributed to cardiovascular causes, equating to around one-third of all global deaths.^1^ Much of this mortality is due to population growth and ageing, and the associated accumulation of risk factors, although the increase in age-standardized rates of cardiovascular disease in some regions suggests that other factors also contribute. Hypertension remains the leading risk factor for cardiovascular disease, affecting over one-quarter of the world’s population.^2^ Additionally, the prevalence of chronic kidney disease, type 2 diabetes mellitus, and obesity is increasing and each independently contributes to cardiovascular disease risk.^1^

Endothelial dysfunction is central to the development of cardiovascular disease, promoting inflammation, thrombosis, and the development of arterial stiffness.^3^ Current management of cardiovascular disease focuses on the use of antihypertensive medications, antiplatelet agents, and cholesterol-lowering therapies. Several of these agents improve endothelial function.^3,4^ However, despite these therapies, cardiovascular disease is associated with an unacceptable burden of morbidity and mortality, and there is increasing urgency to find newer treatments. An agent that could provide cardiovascular protection and benefit associated conditions would be particularly attractive.

The apelin system is a broad regulator of physiology. It consists of the apelin receptor (encoded by the *APLNR* gene previously known as *APJ*) and its two endogenous ligands, apelin and elabela (also known as Toddler).^5^ The system is a particularly appealing target for cardiovascular disease as it promotes endothelium-dependent vasodilatation, inotropy, lowers blood pressure, and increases aqueous diuresis. Activating the apelin system also has metabolic and renal benefits. This review will provide an overview of the role of apelin signalling in the (patho)physiology of cardiovascular disease and the potential benefits of apelin treatment. The following should be consulted for a more detailed discussion of apelin agonism in disorders of water balance,^6^ diabetes mellitus,^7^ obesity^8,9^ and metabolic disorders,^10,11^ and apelin analogues and therapeutic agents.^6,12,13^

## 2. The apelin system

### 2.1 Apelin

**Figure 1:**
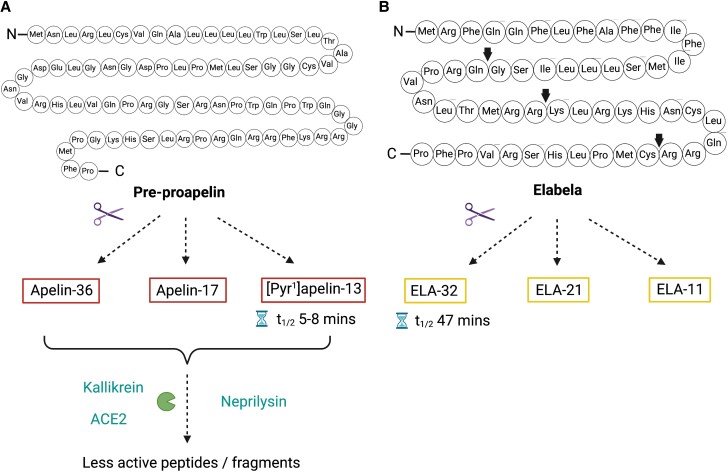
(*A*) Apelin peptides are cleaved from the precursor pre-proapelin. Several circulating isoforms have been identified including apelin-36, apelin-17, and pyroglutamated apelin-13 ([Pyr^1^]apelin-13), the commonest isoform in human plasma. Apelin peptides are subsequently cleaved into less active peptides by plasma kallikrein, angiotensin-converting enzyme 2 (ACE2), and neprilysin. (*B*) Elabela (ELA) is a 54-amino acid peptide with 3 predicted mature isoforms, ELA-32, ELA-21, and ELA-11. Predicted cleavage sites are highlighted by arrows. The half-life of ELA-32 is 10 times that of apelin peptides. Figure created using BioRender.com.

Apelin, encoded by the *APLN* gene on the long arm of the X-chromosome, was the first ligand identified for the apelin receptor in 1998.^14^ Apelin peptides are formed by cleavage of the 77-amino acid precursor, pre-proapelin (*Figure 1* and *Table 1*). Pyroglutamated apelin-13 ([Pyr^1^] apelin-13) is the predominant isoform in the human cardiovascular system and plasma,^15,16^ with other biologically active isoforms including apelin-36, apelin-17, and apelin-13 also detectable.^5^ The half-life of these peptides is short (a few minutes) and may be even shorter *in vivo* than *ex vivo*, a major limitation for clinical application.^17^ Apelin peptides are cleaved by plasma kallikrein, neprilysin, and angiotensin-converting enzyme 2 (ACE2).^18–22^ Interestingly, only neprilysin has been demonstrated to inactivate apelin peptides fully, which may be of significance when considering the benefits of neprilysin inhibition in heart failure.^19^ In humans, apelin mRNA is expressed throughout the vasculature, the central nervous system and in many organs including the heart, lungs, and kidney.^5^ Apelin protein is predominantly found within endocardial and vascular endothelial cells, suggesting that circulating apelin may originate from these tissues.^23^ Centrally derived apelin (e.g. from magnocellular neurons) may also contribute to circulating apelin.^24^ However, plasma apelin concentrations are low so it does not appear to be a major circulating hormone and it may function in an autocrine/paracrine manner.

**Table 1:** Apelin receptor endogenous peptides, and synthetic peptide and small molecule agonists and antagonists

	Action	Value	Parameter
*Human endogenous peptides*			
apelin-13	Full agonist	8.8, 9.2	pIC_50_
[Pyr^1^]apelin-13	Full agonist	8.9	pIC_50_
apelin-17	Agonist	9.0	pIC_50_
apelin-36	Full agonist	8.6	pIC_50_
Elabela/Toddler-11	Agonist	7.2	pIC_50_
Elabela/Toddler-21	Agonist	8.7	pIC_50_
Elabela/Toddler-32	Agonist	8.7	pIC_50_
*Radiolabelled apelin peptide analogues*			
[^3^H](Pyr^1^)[Met(0)11]-apelin-13	Full agonist	8.6	pK_d_
[^125^I]apelin-13	Full agonist	9.2	pK_d_
[^125^I](Pyr^1^)apelin-13	Full agonist	9.5	pK_d_
[^125^I][Glp^65^Nle^75^,Tyr^77^]apelin-13	Full agonist	10.7	pK_d_
*Agonists: peptide analogues*			
cyclo apelin-12 (1–12)	Full agonist	6.3	pEC_50_
cyclourea apelin-12 (1–7)	Full agonist	6.8	pEC_50_
cyclo apelin-12 (7–12)	Full agonist	7.1	pEC_50_
Palmitate-VTLPLWATYTYR (compound 1 [PMID: 25241924])	Full agonist	7.5	pEC_50_
LIT01–196	Agonist	9.1	pK_i_
H2N-c[X-R-L-S-X]-K-G-P-(D-2Nal) (compound 40 [PMID: 34982553])	Agonist	8.2	pK_i_
H2N-c[X-R-L-S-X]-K-G-P-(D-1Nal) (compound 39 [PMID: 34982553])	Agonist	9.2	pK_i_
MM07	Biased agonist	9.5	pEC_50_
*Agonists: small molecules*			
ML233	Full agonist	5.4	pEC_50_
E339-3D6	Agonist	6.4	pK_i_
BMS-986224	Agonist	9.5	pK_d_
Azelaprag (AMG 986; BGE-105)	Agonist	9.5	pEC_50_
Compound 15a [PMID:31724863]	Agonist	10.0	pEC_50_
Compound 21 [PMID: 34855405]	Agonist	10.2	pEC_50_
Compound 14a [PMID: 34795866]	Agonist	10.6	pEC_50_
CMF-019	Biased agonist	10.0	pEC_50_
*Antagonists*			
MM54 peptide	Antagonist	8.2	pK_i_
ALX40-4C peptide	Antagonist	5.5	pIC_50_
ML221 small molecule	Antagonist	5.8	pIC_50_

### 2.2 Elabela

Elabela, the second ligand for the apelin receptor from the *APELA* gene, is a 54-amino acid peptide that was originally identified in the human genome as a potentially secreted peptide in 2013 and provided an elegant explanation for the difference between the phenotypes of apelin and apelin receptor knockout mice.^25,26^ Whilst animals lacking apelin develop normally,^27^ those lacking the apelin receptor show significant cardiovascular developmental defects and many do not survive to birth.^27,28^ Similarly, mice lacking elabela show cardiovascular defects and embryologic lethality. However, there are some differences between the phenotypes early in embryogenesis with ∼10% of elabela mutants showing abnormal yolk sac vasculature with variable cardiac malformations in contrast to only ∼2% of apelin receptor mutants.^29^ Embryos lacking the apelin receptor also show abnormal tail bending. Therefore, it is possible that elabela may also signal through currently unidentified alternative pathways. There are several predicted mature isoforms of elabela peptides, elabela-32, elabela-21, and elabela-11 (*Table 1*).^25,26^ At present, elabela has only been detected in the vascular endothelium and the kidney in adult humans.^30,31^ Of note, the half-life of elabela-32 in human plasma *ex vivo* is 10 times longer than that of apelin.^32^ Intriguingly apelin and elabela interact with different amino acids in the orthosteric binding site of the apelin receptor, although impact on signalling remains to be explored.^33–35^

### 2.3 The apelin receptor

The apelin receptor was first identified in 1993.^36^ It is a G-protein coupled receptor that shares ∼50% homology with the angiotensin II-type 1 receptor (AT_1_) but is not activated by angiotensin II. In fact, the actions of the apelin system broadly oppose those of the renin-angiotensin system.^37–43^ The apelin receptor couples to pertussis toxin-sensitive G_i_ proteins, and binding of either apelin or elabela results in inhibition of adenylyl cyclase and a reduction in intracellular cyclic adenosine monophosphate.^44^ Downstream signalling then occurs via extracellular regulated kinases (ERKs) and phosphoinositide 3-kinase (PI3K)-AKT pathways.^5,44^ The resulting physiological action is dependent on the cell type activated. Proposed apelin and elabela signalling pathways are shown in *Figure 2A*.^45–47^

**Figure 2:**
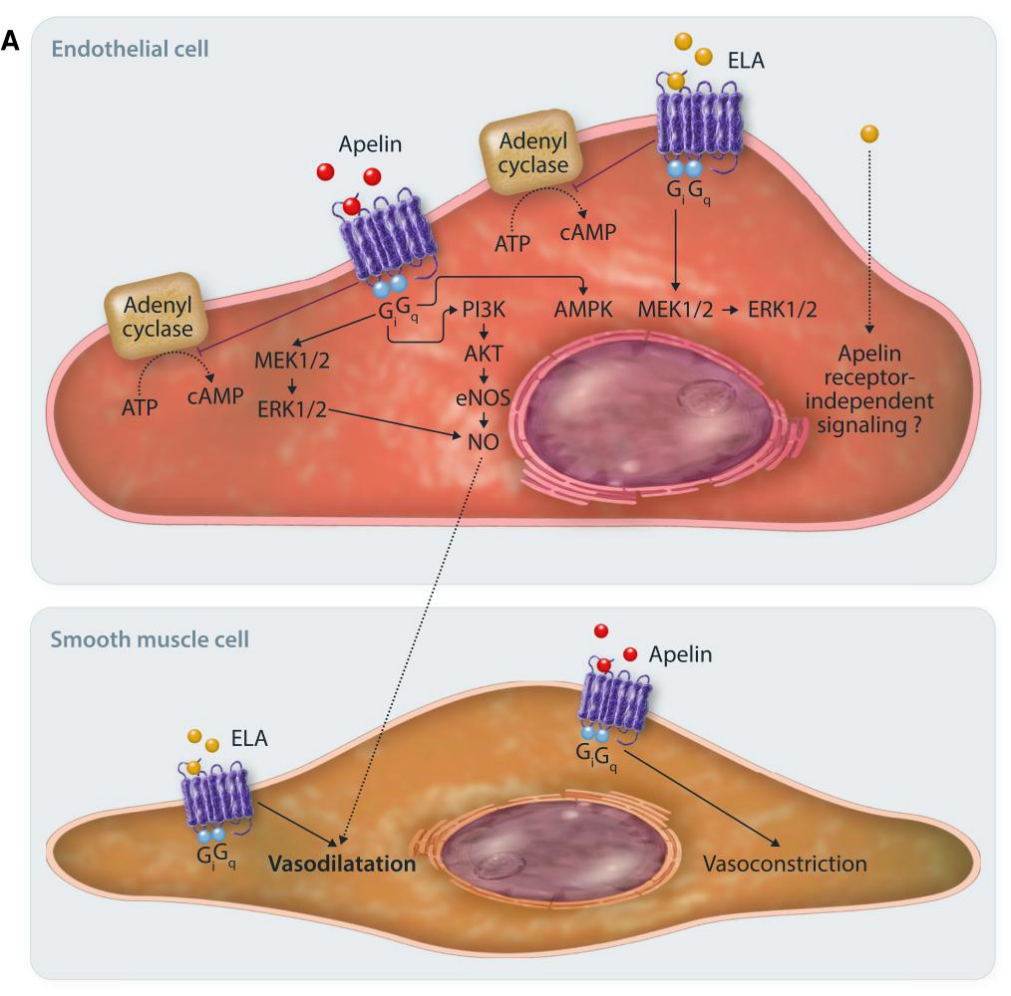
(*A*) Endothelial cell. The apelin receptor couples to pertussis toxin-sensitive inhibitory G proteins (Gi) and ligand binding inhibits adenylyl cyclase and cyclic AMP production and promotes activation of extracellular signal-regulated protein kinase 1/2 (ERK1/2) pathways. Within the vasculature, apelin and elabela promote vasorelaxation through different mechanisms. Apelin promotes nitric oxide (NO) production via ERK1/2, phosphatidylinositol 3-kinase/protein kinase B (PI3K-AKT), and AMP-activated protein kinase (AMPK) pathways. However, when acting on vascular smooth muscle cells, apelin promotes vasoconstriction. Elabela activates ERK1/2 pathways however nitric oxide production is not required for vasorelaxation to occur. Additionally, elabela causes direct vasorelaxation of vascular smooth muscle cells.

**Figure 2:**
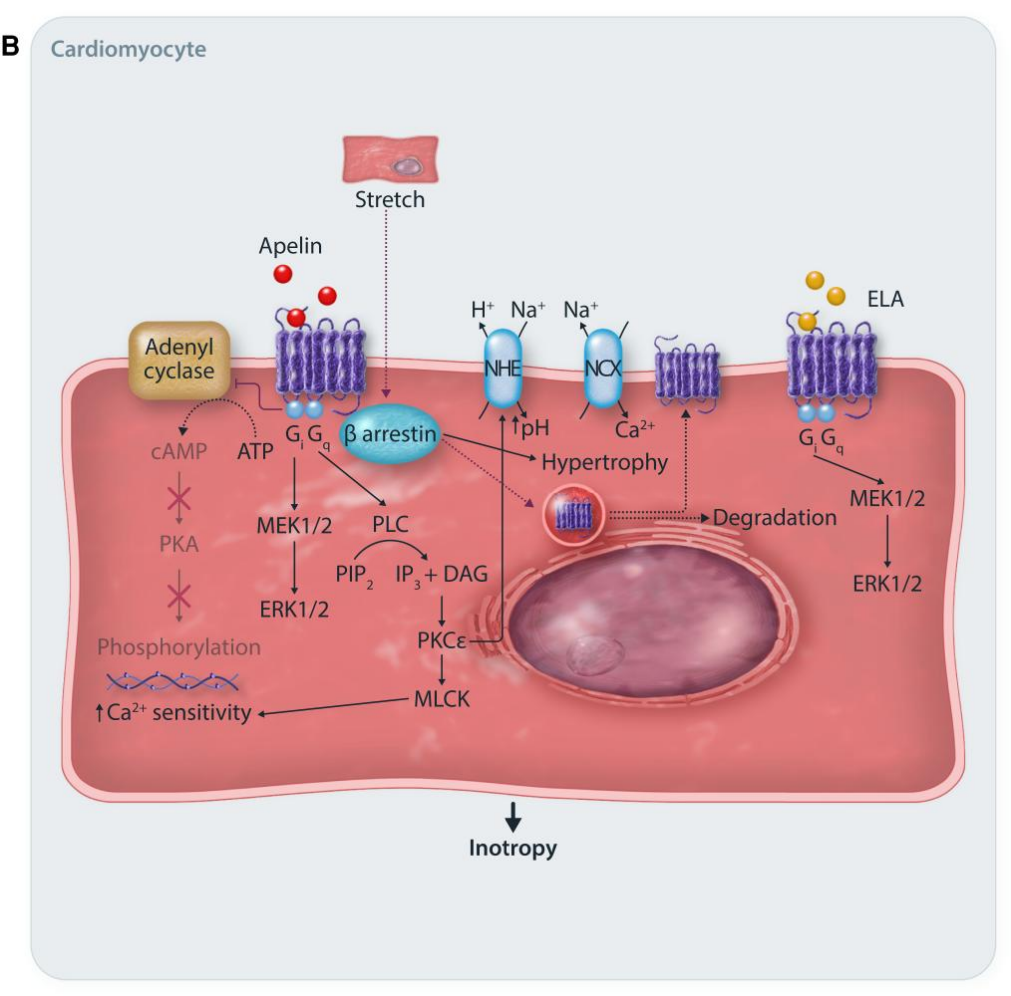
(*B*) Cardiomyocyte signalling. Signalling at the apelin receptor in cardiomyocytes promotes inotropy and an increase in cardiac output through several mechanisms. Gi-protein signalling and inhibition of adenylyl cyclase and the protein kinase A (PKA) pathway, with subsequent inhibition of phosphorylation of troponin and enhanced calcium sensitivity. Apelin also signals via Gi to promote ERK1/2 signalling. Gq-mediated signalling promotes the phospholipase C (PLC)/protein kinase Cɛ (PKC) pathway, stimulating the Na^+^-H^+^ exchanger that ultimately leads to increased intracellular pH. The subsequent increase in intracellular Na^+^ promotes activation of the Na^+^-Ca^2+^ exchanger, increasing intracellular calcium concentration. PKCɛ also stimulates activation of myosin light chain kinase, promoting phosphorylation of myosin and again increasing calcium sensitivity. The unoccupied apelin receptor signals via β-arrestin to promote myocardial hypertrophy, which is inhibited by ligand binding. When the receptor is activated, β-arrestin promotes internalization of the receptor with subsequent recycling to the cell membrane. Elabela has been shown to promote inotropy through ERK1/2 signalling pathways but not PKC pathways.

Following receptor activation, β-arrestins are recruited and the receptor is internalized before being recycled to the cell surface.^5,30,33,48^ Receptor internalization and recycling appear to be dependent on the activating ligand.^48,49^ β-Arrestins are also implicated in ligand-independent signalling at the apelin receptor (*Figure 2B*). Cardiomyocytes develop markers of hypertrophy in response to stretch, and this is dependent on the presence of the apelin receptor. Knockdown of β-arrestins or administration of apelin protects against this.^50^ Thus, when not bound by ligand, the inactivated apelin receptor may act as a mechanosensor that promotes hypertrophy through β-arrestin signalling.

Apelin receptor message is expressed in many tissues including the adipose tissue, heart, lung, kidney, placenta, and skeletal muscle, and also throughout the central nervous system.^5^ Apelin receptor protein is expressed in the brain, heart, kidney, and lung and spinal cord and within the cardiovascular system, it is present throughout the vascular endothelium, in vascular smooth muscle cells of conduit arteries and veins, and in cardiomyocytes.^5^ Similarities in the expression patterns of apelin and the apelin receptor suggest a predominantly autocrine or paracrine mechanism of action.

## 3. The apelin system and the renin-angiotensin system

**Figure 3:**
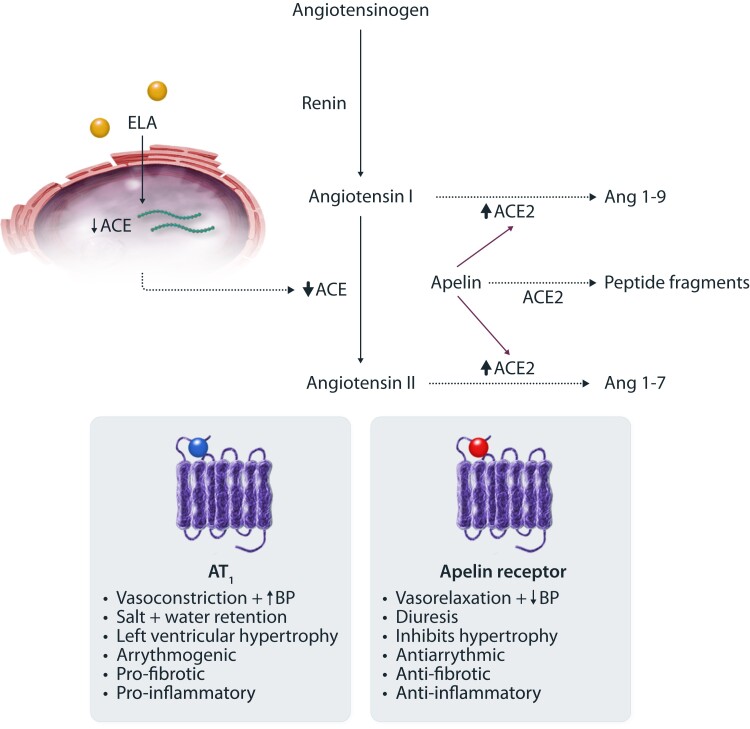
There is crosstalk between the apelin system and the renin-angiotensin system. Angiotensin I is converted to angiotensin II (angiotensin II) by angiotensin-converting enzyme (ACE) and then acts on the angiotensin II-type 1 receptor (AT1) to promote an increase in blood pressure (BP), left ventricular remodelling, inflammation, and fibrosis. Activation of the apelin receptor by apelin or elabela opposes the actions of angiotensin II. The apelin system also influences production of angiotensin II. Elabela reduces expression of ACE, indirectly limiting production of angiotensin II, and apelin promotes production of angiotensin-converting enzyme 2 (ACE2), enhancing breakdown of both angiotensin I and II and promoting the production of angiotensin 1–7 (angiotensin 1–7) that causes vasodilatation.

Overactivation of the renin-angiotensin system is central to the development of cardiovascular disease. Angiotensin II promotes salt retention, hypertension, and end-organ inflammation and fibrosis. Therefore, angiotensin-converting enzyme (ACE) inhibitors and angiotensin receptor blockers are key therapeutic tools in the management of cardiovascular disease. The apelin and AT_1_ receptors are co-expressed throughout the cardiovascular system, and the apelin system opposes the actions of the renin-angiotensin system (*Figure 3*).^10,37,51^ The systems may also reciprocally regulate each other.^52^ In an animal model of heart failure, down-regulation of cardiac apelin and apelin receptor mRNA is restored by AT_1_ blockade. Similarly, angiotensin II infusion down-regulates cardiac apelin mRNA that is restored by an AT_1_ blocker.^38^ Loss of apelin potentiates angiotensin II-induced myocardial injury and abdominal aortic aneurysm development, and apelin treatment reverses these changes.^18,42,43,53^ Angiotensin-converting enzyme 2 (ACE2) is a major negative regulator of angiotensin II, converting it to angiotensin 1–7 that promotes vasodilatation.^54,55^ ACE2 also cleaves apelin peptides to generally less active compounds, and its production is enhanced by them.^10,22,56^ Elabela does not affect ACE2 but down-regulates ACE expression.^41^ If apelin analogues could act synergistically with renin-angiotensin system blockers to offer broad cardiovascular benefits, this would be a particularly useful therapeutic development.

## 4. Clinical targeting of the apelin system

**Figure 4:**
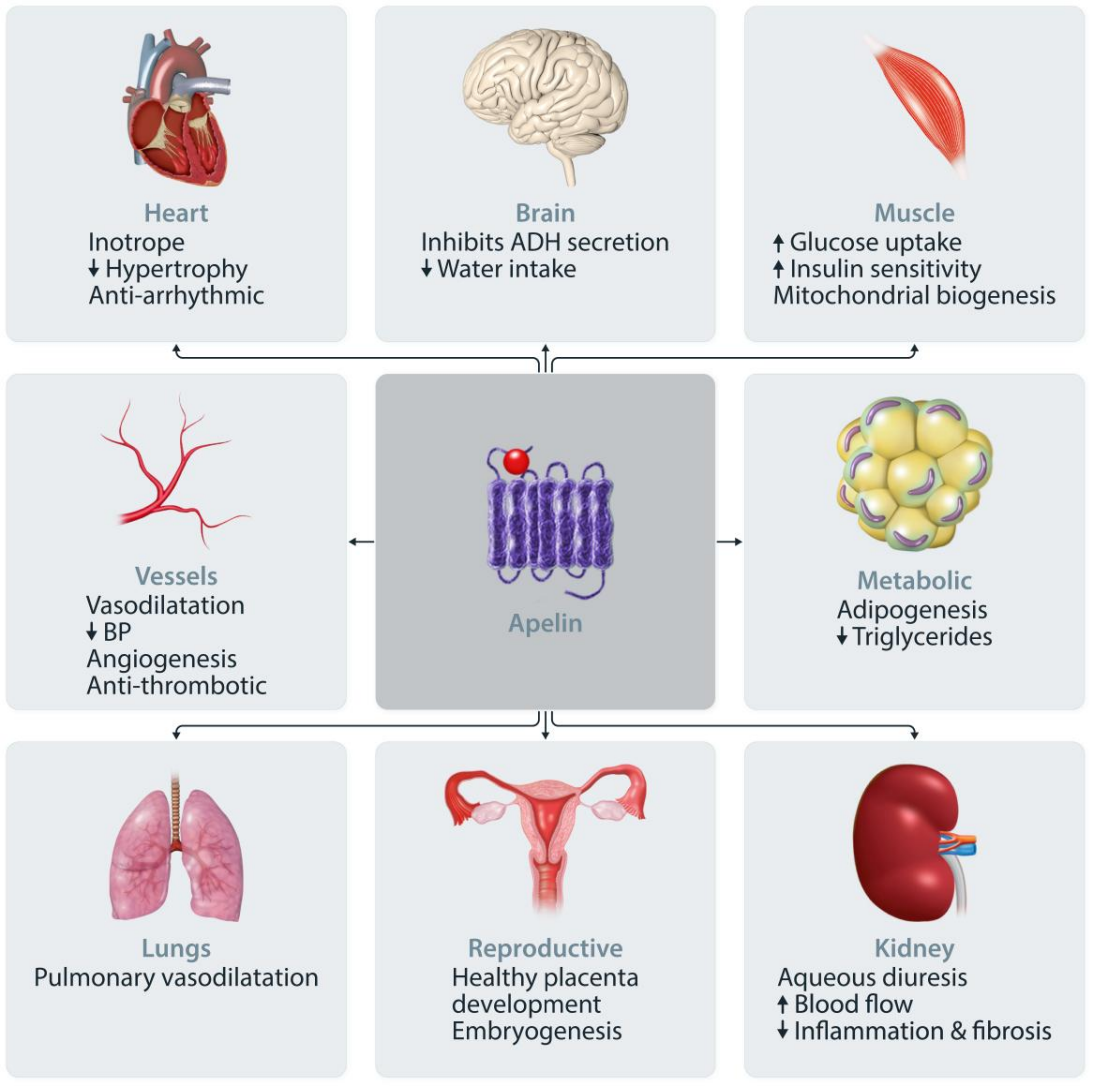
The apelin system is a broad regulator of physiology.

Although several clinical studies have examined the actions of the apelin system in health and disease, all have focused on apelin peptides given the relatively recent discovery of elabela. Overall, the apelin system offers exciting therapeutic potential for a range of cardiovascular diseases (*Figure 4*).

### 4.1 Hypertension

Hypertension, recently re-defined by the *American Heart Association* as a systolic blood pressure ≥ 130 mmHg or diastolic blood pressure ≥ 80 mmHg, is a leading cause of cardiovascular disease and affects almost 50% of adults in the USA.^57^ Many adults require a combination of antihypertensive agents, but despite this, only ∼45% adults achieve blood pressure control.^58^

The apelin system regulates vascular tone *in vitro*, *ex vivo*, and *in vivo*. Activation of the apelin receptor on vascular endothelial cells by apelin peptides promotes nitric oxide production and leads to vasodilatation, with ß-arrestin recruitment also implicated.^10,37^ This occurs in healthy and diseased vessels although vasodilatation may occur via prostanoids rather than nitric oxide in diseased states.^59,60^ In contrast, several studies do report a vasoconstrictor action of both central and peripherally administered apelin *in vitro* and *in vivo* however these involve different animal species and different vascular beds.^61^ Elabela causes dose-dependent vasodilatation, both in the presence and absence of endothelium, but is not dependent on nitric oxide.^31,45^*In vivo* studies find that elabela attenuates angiotensin II-induced increases in blood pressure.^30,41^

In hypertensive rats, apelin receptor message and protein are reduced in the aorta, heart, and kidney, and elabela mRNA is reduced in the renal medulla.^62–64^ The development of hypertension is accelerated by elabela deficiency, and both apelin and elabela are protective against it.^37,64^ Overexpression of elabela also reduced kidney injury and fibrosis through inhibition of the Nod-like receptor protein 3 inflammasome, and, intriguingly, in this study, elabela appeared to be acting independently of the apelin receptor.^64^ Similarly, in the Dahl salt-sensitive rat, overexpression of elabela in the heart delayed the onset of hypertension and was protective against kidney damage.^65^ Of interest, fractional excretion of sodium and chloride was reduced both before and after the initiation of a high-salt diet in these animals, despite no obvious change in expression of elabela within the kidney. Unexpectedly, cardiac function also deteriorated that the authors hypothesized may be due to sodium resorption.^65^ In the hypertensive DOCA-salt rat, the long-acting apelin analogue LIT01-196 significantly reduced blood pressure with once-daily dosing, without inducing any undesirable change in kidney function or sodium concentration.^66^

Clinical studies in healthy volunteers confirm apelin-induced vasodilatation to be dependent on nitric oxide.^17^ Systemic infusion of [Pyr^1^]apelin-13 leads to a ∼5% reduction in blood pressure and a fall in peripheral vascular resistance in health and heart failure.^39,67^ Importantly, apelin promotes vasodilatation even in the setting of renin-angiotensin system activation,^39^ and circulating concentrations of apelin are lower in patients with hypertension.^68–70^ There is no clinical evidence of apelin-induced vasoconstriction in health or disease.^17,39,67,71,72^

Overall, the apelin system is altered in hypertension and is a potential therapeutic target. Apelin and elabela promote vasodilatation by different mechanisms and could have varying roles in maintaining vascular health. Further pre-clinical and clinical studies are required to further define these mechanisms and determine the actions of elabela on the vasculature in humans.

### 4.2 Atherosclerosis

Atheromatous cardiovascular disease is the leading cause of mortality worldwide. The role apelin plays in the pathogenesis of atherosclerosis has not been fully defined. It promotes vascular smooth muscle cell proliferation,^73^ and loss of the apelin receptor protects against the development of atherosclerosis in the apolipoprotein E knockout mouse.^74^ However, loss of apelin promotes atherosclerosis in this model and apelin treatment reduces angiotensin II-induced atherosclerosis^43^ and enhances atherosclerotic plaque stability.^75^ The reasons for these discrepancies are unclear, and to date, there is no evidence for ligand-independent signalling at the apelin receptor in this setting.

In humans, apelin expression is up-regulated within atherosclerotic coronary artery, and both apelin and the apelin receptor are found in atherosclerotic plaques, co-localizing with macrophages and smooth muscle cells.^76^ It is unclear whether this is protective—perhaps through opposition of angiotensin II signalling—or pathological. Patients with coronary artery disease have lower circulating apelin concentrations, and these are lowest in those with symptomatic coronary artery disease.^77^

The relationship between statin use and apelin is intriguing. Statins lower low-density lipoprotein cholesterol but also have pleiotropic effects that contribute to their beneficial cardiovascular effects.^78^ Statins induce kruppel-like factor 2, an important regulator of endothelial cell homeostasis, and subsequently production of endothelial nitric oxide synthase and thrombomodulin.^79^ This promotes vasorelaxation alongside anti-inflammatory and antithrombotic effects, and is dependent on an intact apelin signalling pathway.^80^ Statins also reduce monocyte adhesion to endothelial cells, and this is impaired by loss of apelin. Additionally, statins promote endothelial expression of the apelin receptor at the mRNA and protein level,^80^ and increase circulating apelin peptide concentrations.^81^ There is, therefore, potential for synergistic vascular benefit from combined statin and apelin therapy in patients with atherosclerotic disease. However, this is yet to be conformed in clinical studies.

### 4.3 Myocardial infarction

Atherosclerotic plaque rupture is the first step in the cascade of events that leads to thrombotic coronary artery occlusion and myocardial infarction. Both apelin and the apelin receptor are expressed on human platelets, and apelin can inhibit platelet aggregation through the nitric oxide-cyclic guanosine monophosphate pathway.^82^ Apelin-deficient mice have reduced tail bleeding time and enhanced platelet aggregation and are prothrombotic, whereas apelin-13 prolongs tail bleeding in wild-type animals.^82^ Apelin can also influence thrombosis by regulating plasminogen activator inhibitor-1 (PAI-1) that inactivates the endothelium-derived fibrinolytic factor, tissue plasminogen activator. PAI-1 expression is induced by angiotensin II^83^ but inhibited by apelin.^40^ No clinical studies have explored whether apelin has antithrombotic actions in humans. Current medical management of myocardial infarction involves treatment with dual antiplatelet agents. The potential for apelin to offer additional antiplatelet activity and broad anticoagulant effect is an attractive prospect that deserves further examination.

Following thrombotic occlusion of the coronary artery, tissue ischaemia and myocardial cell death occur, and ultimately an area of scar tissue forms that can lead to left ventricular impairment. The apelin system promotes new vessel formation and can minimize ischaemic injury.^31,84,85^ Hypoxia induces apelin expression *in vitro*, *ex vivo*, and *in vivo*, and this is driven by hypoxia inducible factor 1α.^86–88^ Ischaemia-reperfusion injury is a widely used model of myocardial infarction, and in this model, animals lacking apelin show enhanced susceptibility to injury with larger infarct sizes, more significant left ventricular impairment and an increase in mortality.^89^ Following injury, apelin and apelin receptor mRNA and protein are up-regulated initially, then down-regulated as early as 24 h on. Apelin and elabela both protect against ischaemia-reperfusion injury in models, including when given at the time of reperfusion mimicking potential clinical utility.^86,89,90^ Their beneficial effects are likely due to the promotion of angiogenesis, activation of the PI3K-AKT and p44/42 mitogen-activated protein kinase components of the reperfusion injury salvage kinase pathway and, with respect to apelin, nitric oxide production.^89–93^

Following myocardial infarction, the renin-angiotensin system is activated, and this propagates myocardial injury. Thus, renin-angiotensin system inhibition is part of current standard of care treatment. Pre-clinical studies show that pre-treatment with a combination of apelin and the AT_1_ blocker, losartan, results in synergistic benefit following ischaemia-reperfusion injury, reducing infarct size by ∼50% and improving left ventricular function.^94^

No clinical studies have examined the apelin system around the time of myocardial infarction. The available pre-clinical data suggest that apelin receptor agonism could limit myocardial injury and left ventricular impairment and offer broad anticoagulant effects, with additive benefits to current standard of care. Clinical trials of apelin or elabela treatment post-myocardial infarction are needed but are currently impractical due to the lack of oral preparations.

### 4.4 Heart failure

Heart failure is common, with a prevalence of ∼2% in many parts of the Western World.^95^ It is characterized by the progressive loss of cardiac systolic and diastolic function, often with acute exacerbations requiring hospitalization, and is associated with severe morbidity and mortality.^95^ The apelin system has an important role in maintaining cardiac function in health, and is altered in heart failure and may therefore have potential as a beneficial treatment in this setting.

Apelin is the most potent inotrope in isolated human heart tissue discovered to date.^59^ Mice lacking apelin or the apelin receptor show impaired myocardial contractility at baseline, and apelin-deficient animals are more sensitive to the negative effects of ageing and pressure overload.^27,96^ Traditional inotropes increase intracellular calcium concentrations and subsequently myocardial oxygen demand, promote hypertrophy and arrhythmias, and are associated with increased mortality.^97^ The mechanisms of apelin-induced inotropy are not fully understood. There is evidence both for and against apelin increasing intracellular calcium transients,^98–100^ although apelin’s inotropic effect may be mediated by enhanced calcium sensitivity rather than a change in total intracellular calcium. This would suggest that apelin may not be associated with the same adverse effect profile as other inotropes.

Apelin improves contractility via phospholipase C, protein kinase C, and ERK1/2 pathways.^46,47^ It promotes downstream activation of myosin light chain kinase and inhibits protein kinase A-induced phosphorylation of troponin I, both of which enhance myofilament calcium sensitivity (*Figure 2B*).^47,100^ Elabela also promotes dose-dependent inotropy in isolated perfused rat hearts, and this is partly via ERK1/2 pathways.^34,45^*In vivo*, chronic apelin infusion increased cardiac output without causing myocardial hypertrophy.^101^ Similarly, clinical studies show that intracoronary apelin-36 increased left ventricular contractility, and both short and prolonged systemic infusions of [Pyr^1^]apelin-13 increased cardiac index whilst lowering blood pressure and peripheral vascular resistance.^39,67^

The apelin system is implicated in the pathogenesis of heart failure, with expression of apelin and its receptor up-regulated early in the disease and down-regulated as it progresses.^102–104^ Apelin protein has been detected in myocardial cells from patients with severe heart failure, yet is not found in these cells in health.^103^ Importantly, in patients with heart failure, *APLNR* was the most up-regulated gene in the left ventricle following placement of a left ventricular assist device and was associated with up-regulated tissue levels of apelin protein.^103^ Plasma apelin concentration increases in early heart failure, falls with disease progression, and is restored by cardiac resynchronization therapy.^102,105^ Overall, it may be that early on in disease, the apelin system is attempting to support the failing heart but is less able to do so as cardiac function deteriorates.

Pre-clinical studies find that apelin and elabela are protective in models of heart failure, improving cardiac contractility, preventing hypertrophy, and reducing mortality.^41,89,106,107^ Their actions may be mediated through regulation of the renin-angiotensin system. Apelin acts to inhibit the detrimental actions of angiotensin II *in vitro* and *in vivo*.^53^ Additionally, treatment with ACE inhibitors restores cardiac function in the apelin knockout mouse,^53^ and in a rodent model of chronic heart failure treatment with an AT_1_ blocker restored cardiac apelin and apelin receptor expression.^38^ Elabela also prevents the pressure overload-induced increase in ACE mRNA and protein, restoring the balance of ACE and ACE2.^41^

Both brief and prolonged infusions of [Pyr^1^]apelin-13 lead to a sustained ∼10% increase in cardiac index, with increased ejection fraction seen on echocardiography, and a reduction in systemic vascular resistance and blood pressure in patients with chronic heart failure.^39,67^ Currently standard of care treatment for heart failure includes combination therapy with a renin-angiotensin system inhibitor, beta blocker, and mineralocorticoid receptor antagonist. Recently, a combination of an angiotensin receptor antagonist and neprilysin inhibitor was shown to improve patient outcomes in patients with reduced ejection fraction and these agents are now recommended for patients who remain symptomatic despite ACE inhibitor therapy.^108^ As neprilysin fully inactivates apelin peptides, the success of these agents might be partly due to an increase in apelin peptide levels and this warrants further investigation in clinical studies.

In end-stage heart failure, patients may undergo heart transplantation. The longevity of the transplant is limited by the development of immune-mediated vascular injury in the graft, and apelin may act to mitigate this. Apelin receptor agonism protects against immune-mediated vascular injury both *in vitro* and in a mouse model of heart transplantation, and apelin is up-regulated in myocardial microvasculature and arteries within human failing heart grafts.^109^ Overall, this may be an attempt to reduce vascular injury and apelin treatment may offer therapeutic promise in the transplant setting.

Overall, a combination of inotropy and vasodilatation has been shown to improve haemodynamics in acute heart failure,^110^ and targeting the apelin system in heart failure could therefore offer benefit over and above that from currently available treatments. Clinical trials of apelin receptor agonism are now needed in both acute and chronic heart failure.

### 4.5 Atrial fibrillation

Atrial fibrillation is the commonest sustained arrhythmia in the general population, and is associated with other cardiovascular diseases and morbidity.^111^ Apelin is involved in regulation of cardiomyocyte electrophysiology, acting on several ion channels to shorten the action potential and increase conduction velocity.^99,112^ Slowed myocardial conduction velocity is associated with an increased risk of arrhythmia,^113^ and apelin knockout mice have reduced atrial conduction velocities.^114^

Apelin protects against the development of atrial fibrillation in pre-clinical studies by prolonging the atrial refractory period and inhibiting the actions of angiotensin II.^114,115^ The apelin system may also contribute to thrombotic risk. In patients with atrial fibrillation and thrombosis, expression of apelin and the apelin receptor was reduced and expression of AT_1_ receptors and PAI-1 was increased in the left atrial appendage compared to those in sinus rhythm or atrial fibrillation without thrombosis.^116^

Both atrial and plasma apelin and plasma elabela concentrations are reduced in patients with atrial fibrillation, even in the presence of other cardiovascular comorbidities.^114,117–119^ Plasma apelin also independently predicts the risk of atrial fibrillation and its recurrence following successful cardioversion or pulmonary vein isolation.^119–121^

Taken together, the apelin system protects against the development of atrial fibrillation by influencing electrical conduction and there is further evidence of its potential antithrombotic actions. Increasing circulating apelin in patients with atrial fibrillation could be a future therapeutic strategy, and apelin may be useful biomarker for atrial fibrillation.

### 4.6 Pulmonary arterial hypertension

Pulmonary arterial hypertension is a rare, chronic, and progressive disorder characterized by pulmonary vascular remodelling with smooth muscle cell hypertrophy, increasing vascular resistance and ultimately right ventricular failure.^122^ It has an estimated incidence of 15–50 cases per million and predominantly affects women.^122^ Pulmonary arterial hypertension may be idiopathic, genetic, or secondary to a variety of causes including drugs (e.g. the appetite suppressant aminorex), infection (e.g. schistosomiasis), or multi-system disease (e.g. connective tissue diseases).^122^ Survival has increased dramatically in the last two decades from a 5-year survival of 34% due to newer therapies becoming available. However, there is still substantial associated morbidity and mortality, and prognosis is dependent on many factors including aetiology and the severity of disease.^122^ Despite current treatments, patients have an overall 3-year survival of only 83%.^123^

Apelin, elabela, and the apelin receptor are expressed throughout the pulmonary vasculature.^5^ A small interfering RNA approach to induce apelin deficiency in pulmonary artery endothelial cells impaired cell survival and promoted pulmonary artery smooth muscle cell hypertrophy. Conversely, apelin treatment of pulmonary artery endothelial cells protected against cell death and promoted pulmonary artery smooth muscle cell apoptosis.^124^ Expression of the system is altered in models of pulmonary arterial hypertension, with reduced apelin, elabela, and apelin receptor expression in the right ventricle, and this may contribute to its pathogenesis.^30,125^ Mice lacking the apelin gene develop worse pulmonary arterial hypertension in response to hypoxia than wild-type animals, with reduced endothelial nitric oxide synthase and more pronounced vascular remodelling.^126^ Importantly, animal models of pulmonary arterial hypertension show that treatment with apelin, the G-protein-biased apelin receptor agonist MM07, or elabela can improve pulmonary haemodynamics and even reverse pulmonary arterial hypertension.^30,124,125,127^

Patients with pulmonary arterial hypertension have dysfunctional pulmonary artery endothelial cells, and these exhibit reduced apelin and elabela expression.^30,124,128^ Plasma apelin concentrations are reduced in patients with pulmonary arterial hypertension,^126^ and short infusions of [Pyr^1^]apelin-13 led to additional favourable changes in pulmonary haemodynamics, with a fall in pulmonary vascular resistance and a rise in cardiac output.^72^ Interestingly, although this study was not designed to explore this effect, patients on phosphodiesterase-5 inhibitors with more severe disease showed a greater improvement in pulmonary vascular resistance, stroke volume, and cardiac output than those patients not on this treatment.^72^ Phosphodiesterase-5 inhibitors act on the nitric oxide pathway, preventing the degradation of cyclic guanosine monophosphate and thereby promoting vasodilatation. Apelin may therefore offer synergistic benefit in this setting by promoting upstream nitric oxide production.

Mutations in the bone morphogenic protein receptor type 2 (BMPR2) gene and alterations in BMPR2 signalling can predispose to pulmonary vascular remodelling and the development of pulmonary arterial hypertension.^129^ This may be partly due to regulation of the apelin system. A mouse model of hypoxia-induced pulmonary arterial hypertension found that loss of bone morphogenic protein 9 (BMP9, a ligand of the Activin Receptor-like Kinase 1 receptor that heterocomplexes with BMPR2) increased apelin expression and reduced susceptibility to pulmonary arterial hypertension.^130^ Similarly, application of BMP9 to pulmonary artery endothelial cells from patients with pulmonary arterial hypertension and healthy controls led to reduced apelin expression.^130^ Notably, a pre-clinical model has recently shown that oestradiol is protective against right ventricular failure by up-regulating apelin via oestrogen receptor alpha/BMPR2 signalling.^131^

Taken together, these data suggest that loss of apelin may contribute to the pathogenesis of pulmonary arterial hypertension and apelin treatment may provide additional benefits on top of standard of care. Clinical studies with prolonged apelin treatment and apelin analogues with longer half-lives are needed.

### 4.7 Pre-eclampsia

Pre-eclampsia is a multi-system disorder that affects ∼5% of pregnancies.^132^ It is characterized by the development of gestational hypertension with additional evidence of end-organ dysfunction, such as proteinuria.^133^ It can occur early (<34 weeks’ gestation) or late in pregnancy, and ranges in severity. Once established, there is no curative treatment other than delivery of the foetus, and it remains a major cause of maternal and infant morbidity and mortality.^133^ The pathogenesis of pre-eclampsia is not fully understood, but poor development of the placental vascular network is key, resulting in placental ischaemia.^133^

Apelin and elabela contribute to placental development and embryogenesis, and are implicated in the pathogenesis of pre-eclampsia.^134^ Apelin promotes small vessel angiogenesis and elabela is essential for endoderm differentiation and heart development, and also increases trophoblast invasion into the maternal uterine wall seemingly independent of the apelin receptor.^26,135^ Apelin and pre-proapelin are abundantly expressed in the healthy human placenta,^136^ with [Pyr^1^]apelin-13 the commonest isoform.^137^ Changes in the placental expression of the apelin system in humans with pre-eclampsia are not fully defined. Apelin mRNA and protein and apelin receptor protein are down-regulated in severe disease, but it is unclear whether changes occur earlier.^138,139^ The relationship with the renin-angiotensin system is again implicated. In pre-eclampsia, there is up-regulation of angiotensin II and the AT_1_ receptor.^140^ Angiotensin II reduces apelin release from healthy human chorionic villus explants, but this is prevented and apelin release is in fact enhanced by the AT_1_ receptor blocker, losartan.^137^ Elabela expression has only been explored in the early stages of pre-eclampsia, and no changes are seen.^139^ Interestingly, elabela-deficient, but not apelin-deficient, mice develop a pre-eclampsia phenotype with small, poorly vascularized placentas, hypertension, proteinuria and glomerular endotheliosis (a hallmark feature of pre-eclampsia), and treatment with elabela prevented these changes.^141^ However, apelin treatment does lower blood pressure and proteinuria in models of pre-eclampsia.^142–144^

Published data describing plasma concentrations of apelin and elabela in pre-eclampsia are conflicting and derive from studies with heterogeneous designs and populations that are difficult to compare. On balance, plasma apelin appears to increase in early-onset and severe pre-eclampsia, but whether this is the case in late-onset or mild disease is unclear.^138,139,145^ However, the placenta may not be the source of circulating apelin, so any rise in plasma concentrations may reflect a compensatory increase in production from other tissues due to hypertension. Data on plasma elabela in pre-eclampsia are inconsistent, with both increased and decreased concentrations found.^146–148^ Overall, further studies are required to develop understanding of the apelin system in pre-eclampsia and establish whether it could be a therapeutic target for this disease.

### 4.8 Metabolic disease

The apelin system has a role in glucose and lipid metabolism and may be a therapeutic target for obesity and type 2 diabetes mellitus. A full review of apelin’s metabolic effects has been described recently (see tan-Laurell *et al.*^149^). Briefly, apelin and the apelin receptor are present on adipocytes and pancreatic islet cells.^5,150^ Expression of apelin by these cells is regulated by both insulin and glucocorticoids.^150,151^ Apelin also has a biphasic effect on insulin, with lower concentrations inhibiting and higher concentrations stimulating insulin secretion.^150,152^ Animals lacking apelin are hyperinsulinaemic and show reduced insulin sensitivity.^153^

In healthy mice, apelin infusion lowers plasma glucose by promoting glucose uptake in skeletal muscle and adipose tissue via endothelial nitric oxide synthase, AMPK, and Akt-dependent pathways. This glucose-lowering effect is also seen in obese and insulin-resistant mice,^154^ and is maintained with chronic apelin treatment where these animals showed reduced fat mass, triglycerides, and lower insulin levels than controls.^155,156^ Chronic apelin treatment also inhibits hepatic steatosis.^157^ Apelin analogues also promote glucose uptake and inhibit food intake in healthy and obese mice.^158^ Chronic treatment with apelin-13 analogues improved glycaemia, increased plasma insulin, and improved response to glucose tolerance tests as effectively as the established incretin therapies liraglutide and exendin-4. Interestingly, the apelin analogue (pGlu)apelin-13 amide was more effective at lowering triglyceride levels than the incretin mimetics.^159^ Clinical studies in healthy overweight men also find that apelin enhances insulin sensitivity.^160^ Studies exploring the ability of apelin to influence glycaemia and vascular health in subjects with increased weight and type 2 diabetes mellitus are in progress (see clinicaltrials.gov: NCT03449251).^161^

### 4.9 Kidney disease

Chronic kidney disease is increasing common worldwide and is independently associated with cardiovascular disease.^162^ Indeed, cardiovascular disease is the commonest complication of chronic kidney disease.^162^ The apelin system is a promising therapeutic target in a range of kidney diseases.^6,163^ Apelin regulates glomerular haemodynamics, opposing the actions of angiotensin II at the afferent and efferent glomerular arterioles through nitric oxide production.^52^ The apelin system also contributes to the regulation of fluid balance, acting in opposition to the vasopressin system.^52,164–166^ Within the central nervous system, apelin and the apelin receptor colocalize with vasopressin and the vasopressin 1 receptor within the magnocellular neurons of the hypothalamus.^6^ Apelin promotes aquaresis both by central actions and by directly inhibiting vasopressin-induced insertion of aquaporin 2 channels in the principal cells of the collecting duct.^165,167,168^ Changes in plasma osmolality cause opposing effects on the regulation of vasopressin and apelin release,^16^ and the apelin system may therefore represent a target in disorders of water balance such as syndrome of inappropriate antidiuretic hormone. In a model of vasopressin-induced hyponatraemia, a peptide agonist with a longer half-life than apelin (LIT01-196) blocks the antidiuretic effect of vasopressin and the vasopressin-induced increase in urinary osmolality.^168^ Elabela has also been shown to have an aquaretic effect.^34,164^

Both apelin and elabela are protective in models of acute kidney injury, with anti-inflammatory, anti-apoptotic, and anti-fibrotic effects,^169–171^ that may be synergistic.^171^ At present, there are no published data on the renal actions of the apelin system in humans, but studies are underway (see clnicaltrials.gov: NCT03956576).^172^ A detailed review of the apelin system in kidney disease may be found at Chapman *et al*.^163^

## 5. Therapeutic targeting of the apelin system

Whilst the apelin system offers exciting therapeutic potential for many cardiovascular diseases, future clinical studies are limited by the lack of orally available long-acting compounds. As such, development of apelin analogues resistant to peptidases and small molecule apelin receptor agonists has been a priority over the last decade, with variable success. Apelin peptide modification has been performed by PEGylation, cyclization, the addition of unnatural amino acids and conjugation to domain antibodies that then bind to albumin *in vivo*. The benefits and limitations of currently available agents have been discussed.^10,11^ Some analogues have prolonged half-lives and preserved activity *in vivo*.^66,158,168,173–176^ Several have shown beneficial effects in disease models. One apelin analogue was protective against myocardial injury.^89^ Others promote insulin-dependent glucose lowering in diet-induced-obese mice.^158,177^ As discussed, long-term treatment of diabetic *db/db* mice with two the apelin analogues ((pGlu)apelin-13 amide or pGlu(Lys^8^GluPAL)apelin-13 amide) was in some respects more effective than established incretin therapies at improving metabolic dysfunction.^177^

Given the current understanding of apelin receptor signalling, the ideal agent would be a biased agonist that promotes signalling through G-protein pathways and limits β-arrestin signalling. MM07, a cyclic apelin analogue, is one such biased agonist with a half-life *ex vivo* that is ∼seven-fold greater than [Pyr^1^]apelin-13. Studies in humans have demonstrated it to be a more potent vasodilator than [Pyr^1^]apelin-13 with no evidence of desensitization, and it improved haemodynamics and vascular remodelling in a pre-clinical model of pulmonary arterial hypertension.^71,127^ The small molecule compound CMF-019 also offers biased agonism at the apelin receptor, which is preserved *in vivo*.^178^ Encouragingly, pre-clinical studies not only confirm similar vasodilator and inotropic actions to [Pyr^1^]apelin-13 but it also has potential as a disease-modifying agent in pulmonary arterial hypertension.^178^

Significant effort has been devoted to developing alternative oral small molecule apelin agonists. Two such long-acting agents that have shown promise in pre-clinical studies have undergone phase I trials.^179–181^ Whilst data are as yet unpublished regarding BMS-986224, AMG 986 was shown to be safe and well tolerated in healthy humans and those with heart failure, although data were inconsistent regarding its clinical efficacy.^179,182^ So far, neither agent has progressed to further studies. The search for other small molecule agonists continues, and recently a small molecule known as compound 47 has been shown to be a potent and selective apelin receptor agonist.^183^

## 6. Conclusions

The apelin system is a promising therapeutic target for a range of cardiovascular diseases. There is strong evidence for benefit of apelin receptor agonists in pulmonary arterial hypertension and chronic kidney disease with further potential in heart failure as apelin promotes inotropy and has anti-fibrotic and antiplatelet actions. The precise role of elabela in relation to the apelin signalling pathway in cardiovascular disease remains to be elucidated, particularly whether elabela has distinct physiological, pathophysiological, and pharmacological actions from apelin. Elabela has a longer half-life than apelin and may activate different downstream pathways. Small molecule apelin compounds, including biased agonists such as CMF-019,^178^ are being developed that will enable meaningful clinical studies in this space in the future.
